# Incidence des accidents vasculaires cérébraux chez les patients VIH positifs sous traitement antirétroviral au long court

**DOI:** 10.11604/pamj.2016.24.45.8013

**Published:** 2016-05-11

**Authors:** Yacouba Njankouo Mapoure, Ines Nepetsoun Nkongni, Henry Namme Luma, Bertrand Hugo Mbtachou Ngahane, Esther Barla, Samuel Ngwane, Albert Soné Mouelle, Alfred Kongnyu Njamnshi

**Affiliations:** 1 Faculté de Médecine et des Sciences Pharmaceutiques, Université de Douala, Hôpital Général de Douala, Cameroun; 2 Service d'Hépato-gastro-entérologie, Faculté de Médecine et des Sciences Biomédicales, Université de Yaoundé 1, Cameroun; 3 Service de Pneumologie, Faculté de Médecine et des Sciences Pharmaceutiques, Université de Douala, Hôpital Général de Douala, Cameroun; 4 Service de Pédiatrie, Centre de Traitement Agréé de l'Hôpital Général de Douala, Cameroun; 5 Service de Dermatologie, Hôpital Général de Douala, Cameroun; 6 Département des Sciences Cliniques, Faculté de Médecine et des Sciences Pharmaceutiques, Université de Douala, Cameroun; 7 Département de Neurologie, Hôpital Central de Yaoundé, Faculté de Médecine et des Sciences Biomédicales, Université de Yaoundé 1, Cameroun

**Keywords:** Accidents vasculaires cérébraux, infection à VIH, trithérapie antirétrovirale, Strokes, HIV infection, antiretroviral triple therapy regimen, Cameroon

## Abstract

**Introduction:**

Avec l'introduction de la trithérapie dans les années 1996, la morbidité et la mortalité liées à l'infection par le VIH a nettement diminué. Concomitamment avec ce succès clinique, plusieurs changements métaboliques incluant diabète, hypertension artérielle, dyslipidémie et lipodystrophie ont été observés, ceux-ci étant des pourvoyeurs d'accidents vasculaires cérébraux. L'objectif de ce travail était de déterminer l'incidence des accidents vasculaires cérébraux chez les patients VIH positifs sous traitement antirétroviral au long court.

**Méthodes:**

Il s'agissait d'une étude de cohorte rétrospective, menée dans le Centre de Traitement Agrée de l'Hôpital Général de Douala (HGD), avec un recueil des données sur 10 ans allant de Mai 2001 à avril 2010, portant sur les patients VIH positifs adultes sous traitement antirétroviral depuis au moins 6 mois. Les patients étaient suivis selon le protocole national de prise en charge du Cameroun. La survenue d'un AVC a été déterminée par la méthode Kaplan-Meyer tandis que les facteurs associés à la survenue d'un AVC ont été recherché par le test de Khi-2. Le seuil de signification statistique était fixé à 0,05.

**Résultats:**

307 patients étaient inclus dont 62,4% de sexe féminin, et l’âge moyen était de 40,1 ± 9,9 ans. L'incidence des AVC était de 1,7% sur 72 mois de suivi sans différence significative entre les femmes et les hommes (P= 0,76). Le taux d'incidence calculé était de 0,3 pour 100 personnes années. Dans 85,7% des cas il s'agissait d'un AVC ischémique. Le délai moyen de survenue d'un AVC était de 33,4 mois. Les facteurs associés à la survenue d'un AVC étaient: les patients ayant initié leur traitement au stade III et IV de l'OMS et le taux de CD4 > 100/mm3 à l'initiation du traitement antirétroviral.

**Conclusion:**

L'incidence des AVC chez les patients VIH positifs sous traitement antirétroviral est similaire à celle rapportée antérieurement mais ces AVC surviennent à un âge précoce et justifie une surveillance accrue par les cliniciens prenant en charge ces patients. Une étude prospective avec une population témoins est nécessaire.

## Abstract (en)

**Introduction:**

With the introduction of triple therapy regimen in 1996, the morbidity and mortality associated with HIV infection decreased significantly. Concomitantly with this clinical success, several metabolic changes including diabetes, arterial hypertension, dyslipidemia and lipodystrophy were observed, being associated with the risk of stroke. This study aims to determine the incidence of strokes in HIV-positive patients treated with long term antiretroviral treatment.

**Methods:**

This was a retrospective cohort study of HIV-positive adult patients receiving long term antiretroviral treatment for at least 12 months. It was conducted at the Approved Treatment Centres of Douala General Hospital (HGD); data collection spanned a 10-year period from May 2001 to April 2010. Patients were treated according to Cameroon national treatment guidelines. The Kaplan-Meyer method was used to determine the time of stroke onset. The Khi-2 test was used to investigate the association between two qualitative variables. The statistical significance level was set at 0.05.

**Results:**

407 patients were included in the study, 62.4% were female, the average age was 40.1 ± 9.9 years. The incidence of strokes was 1.7% over 72 months follow up with no significant difference between women and men (P = 0.76). The incidence rate was calculated to be 0.3 per 100 person years. 85.7% of cases had an ischemic stroke. The mean time from stroke onset was 33.4 months. Factors associated with stroke occurrence were: patients who underwent treatment at WHO Clinical Stage III and IV and with CD4 count> 100/mm3at the start of antiretroviral therapy.

**Conclusion:**

The incidence of stroke in HIV-positive patients treated with long term antiretroviral therapy is similar to that previously reported but these strokes occur at an early age and justify increased clinical monitoring. A prospective study involving a control population is needed.

## Introduction

L'infection par le virus de l'immuno-déficience humaine (VIH) demeure un problème de santé publique dans le monde. Au Cameroun, sa prévalence est estimée à 4,2% en 2011 [1]. Depuis l'avènement de la trithérapie antirétrovirale (TAR), on a noté une diminution de la mortalité et la morbidité liées à l'infection par le VIH qui de par son histoire naturelle conduisait la plupart des personnes vivant avec le VIH (PVVIH) à la mort via les infections opportunistes. Concomitamment avec ce succès clinique, plusieurs changements métaboliques incluant diabète, hypertension artérielle, dyslipidémie et lipodystrophie ont été observés, ceux-ci étant des pourvoyeurs d'accidents vasculaires cérébraux. Les manifestations de l'infection à VIH sont polymorphes et le système nerveux n'en est pas épargné. Les manifestations neurologiques sont rencontrées à tous les stades évolutifs de la maladie. Parmi les patients VIH positifs, 75% présententeront une affection neurologique tandis que 90% auront une atteinte neuropathologique [2, 3]. Des études ont mis en exergue la relation qui existe entre infection par le VIH et accident vasculaire cérébral (AVC) et il est actuellement établi que le VIH est un facteur de risque d'AVC [4]. Plusieurs mécanismes expliquent la relation entre l'infection à VIH et les AVC. Ces mécanismes peuvent être directs et dans ce cas le VIH est directement incriminé comme principal facteur de survenue des AVC via des vasculopathies, des coagulopathies ou alors indirects par le biais des maladies opportunistes (méningites chroniques), la consommation des drogues injectables et la prise des thérapeutiques antirétrovirales [4–8]. Les affections neuro-vasculaires et l'infection par le VIH ne sont pas encore étudiées dans le contexte Camerounais. L'objectif de cette étude était de déterminer l'incidence des accidents vasculaires cérébraux chez les patients VIH positifs sous traitement antirétroviral au long court et de rechercher les facteurs associés à leur survenue.

## Méthodes

Il s'agissait d'une étude de cohorte rétrospective ayant concerné les patients venus en consultation de Mai 2000 à avril 2010 au Centre de Traitement Agréé de l'HGD. Nous avions inclus des patients VIH positifs adultes sous traitement antirétroviral depuis au moins 12 mois et suivis régulièrement selon le protocole national de prise en charge des PVVIH au Cameroun. Nous avions pris le délai minimal de 12 mois pour plusieurs raisons: i) éviter les premiers mois du TAR pendant lesquels le patient peut présenter un syndrome de reconstitution immunitaire avec infection neuro-méningée telle qu'une méningo-encéphalite cryptocococcique ou tuberculeuse pouvant entrainer un AVC, ii) après 6 mois, le patient n'est plus censé faire des infections opportunistes sauf en cas d’échec de TAR. Le traitement antirétroviral incluait les combinaisons avec 2 inhibiteurs nucléosidiques et un inhibiteur non nucléosidique ou 2 inhibiteurs nucléosidiques et un inhibiteur de protéase. Nous avions exclu les patients aux antécedants d'AVC avant la mise sous TAR. Pour chaque patient, les données recueillies ont été consignées dans un questionnaire standardisé, pré testé. Les informations requises étaient relatives aux données sociodémographiques, cliniques, biologiques et thérapeutiques. Les dossiers de suivi des patients étaient étudiés tous les 6 mois. Tous les cas d'AVC ont été documentés par le scanner cérébral.

### Considérations éthiques

La confidentialité des données était assurée tout au long du processus et l’étude était approuvée par le Comité National d'Ethique et de la Recherche pour la Santé Humaine au Cameroun (Réf N° 2013/05/270/CNERSH/SP).

### Analyse statistique

L'analyse statistique s'est faite grâce au logiciel SPSS version16 pour Windows. Les tests T de Student et de Fisher ont été utilisés pour comparer respectivement les moyennes et les pourcentages. La méthode Kaplan-Meyer a été utilisée pour déterminer le délai de survenue d'un AVC. Le taux d'incidence d'un AVC a été calculé grâce à la formule suivante: T= n/Δt (n= nombre de nouveaux cas d'accident vasculaire cérébraux, Δt= nombre de personnes-temps) [9]. Le Test de Khi-2 a été utilisé pour rechercher l'association entre deux variables qualitatives. Le seuil de signification statistique était fixé à 0,05.

## Résultats

### Caractéristiques sociodémographiques, cliniques, biologiques et thérapeutiques des patients à l'initiation du traitement antirétroviral (Tableau 1)

Quatre cents sept patients étaient inclus dont 62,4% de sexe féminin pour un sexe ratio F/H de 1,6. L’âge moyen était de 40,1 ± 9,9 ans. La moyenne d’âge des hommes étaient plus élevée que celle des femmes (P = 0,001). Les célibataires représentaient 53% des cas 80,1% résidaient à Douala. 62,4% des patients avaient une activité génératrice de revenus tandis que l'assurance maladie était retrouvée chez 9,3% des patients. La ville de Douala était le lieu de résidence chez 80,1% des patients. La majorité des patients était dépistée pour l'infection par le VIH à l'occasion d'une infection opportuniste (66,6%) dont la principale était la tuberculose pulmonaire avec 13,3% des cas. Le VIH 1 était le plus fréquent rencontré (99,3%). La co-infection VIH et virus de l'hépatite était de 2,5% des cas et concernée plus les hommes (5,2%) que les femmes (0,8%) avec une différence significative (P = 0,005). Et 7 fois sur 10, il s'agissait d'une infection par le virus de l'hépatite B. Le taux moyen de CD4 à l'initiation du TAR était de 167,4 cellules/mm^3^ ± 5,5 cellules/mm^3^ avec des extrêmes allant de 01 à 806 cellules/mm^3^, sans différence significative entre les hommes et les femmes (P = 0,49). Sur 45 patients ayant fait une charge virale au début du TAR, 84,4% avaient une charge virale > = 100.000 copies/mm^3^. La charge virale moyenne était de 311963,7 copies/ml ± 72461,3 copies/ml avec des extrêmes allant de 162 à 2300000 copies/ml. Les combinaisons d'antirétroviraux utilisées étaient stavudine-lamivudine-névirapine (40,3%), zidovudine-lamivudine-efavirenz (29,2%) suivi de la zidovudine-lamivudne-névirapine (13,8%).

**Tableau 1:** Caractéristiques des patients à l'initiation du traitement antirétroviral

Caractéristiques	Effectifs N (%)	Moyenne ± DS (extrêmes)
**Tranches d’âge**		
- Moins de 30 ans	59 (14,5)	-
- 30-39 ans	152(37,3)	-
- 40-49 ans	119(29,2)	-
- Plus de 50ans	77 (18,9)	
**Sexe**		
- Masculin	153(37,6)	-
- Féminin	254(62,4)	-
**Activité génératrice de revenus**		
- Oui	254(62,4)	-
- non	153(37,6)	-
**Assurance maladie**		
- Oui	38 (9,3)	-
- Non	368(90,7)	-
**Circonstances de découverte de la séropositivité**		
- Maladie	271(66,6)	-
- Systématique	136(33,4)	-
Type de VIH		
- VIH1	404(99,3)	-
- VIH1 et 2	3 (0,7)	-
**Coinfection VIH-Hépatites**		
- Oui	10 (2,5)	-
- Non	397(97,5)	-
**Combinaisons de TAR utilisées**		
- D4T/3TC/NVP	164(40,3)	
- AZT/3TC/EFV	119(29,2)	-
- AZT/3TC/NVP	56 (13,8)	-
- D4T/3TC/EFV	31 (7,6)	-
- TDF/3TC/EFV	18 (4,4)	-
- TDF/EMT/EFV	6 (1,5)	-
- Autres	13 (3,2)	-
**Taux de CD4 (/mm3)**	307 (100)	**167,4 ± 5,5**(01-806)
**Charge virale moyenne (copies/mm3)**	45(14,66)	**311963,7 ± 72461,3**(162-2.300.000)

ARV= Antiretroviral, D4T= Stavudine, 3TC= lamivudine, AZT= Zidovudine, EFV= Efavirenz, NVP= Nevirapine, TDF= Ténofovir, EMT= Emtricitabine

### Facteurs de risque cérébro-vasculaire à l'initiation du traitement et incidence des AVC

**Figure 1:**
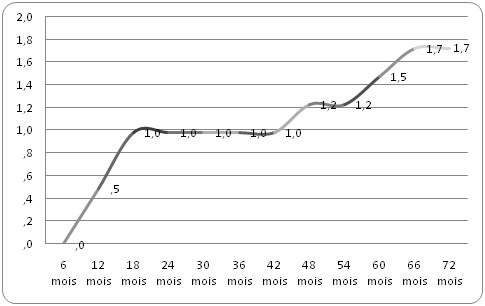
Incidence cumulée des AVC sur 72 mois de suivi

A l'initiation du TAR, les facteurs de risque cérébro-vasculaire étaient l'hypertension artérielle chez 28 (6,9%) patients, le diabète 16 (3,9%) et l'hypercholestérolémie 4 (1%), sans différence significative entre les hommes et les femmes avec respectivement P = 0,32; P = 0,12 et P = 0,62. L'incidence cumulée des AVC était de 1,7% sur 72 mois de suivi. Le taux d'incidence calculé était de 0,3 pour 100 personnes-années avec un délai moyen de survenue de l'AVC à 33,4 mois (Figure 1). L’âge moyen des patients ayant fait un AVC était de 48 ans avec des extrêmes allant de 35 à 60 ans. Les facteurs associés à la survenue des AVC étaient les patients au stade III et IV de la classification OMS et ceux ayant un taux de CD4 à l'initiation du TAR > 100/mm^3^ (Tableau 2). Les AVC étaient ischémiques dans 85,7% des cas contre 14,3% pour les accidents vasculaires cérébraux hémorragiques. LeTableau 3 résume les caractéristiques des patients victimes d'AVC.

**Tableau 2:** Caractéristiques des patients VIH positifs ayant fait un AVC

	Sexe	Âge	CD4 *	OMS	TAR**	Délai***	Type d'AVC
1	Masculin	48ans	271	Stade 2	AZT/3TC/EFV	M 66	Hémorragique
2	Masculin	60ans	53	Stade 4	TDF/3TC/EFV	M 12	Ischémique
3	Féminin	33ans	36	Stade 3	AZT/3TC/NVP	M 12	Ischémique
4	Masculin	55ans	66	Stade 4	D4T/3TC/NVP	M 48	Ischémique
5	Féminin	60ans	11	Stade 4	AZT/3TC/EFV	M 18	Ischémique
6	Féminin	45ans	20	Stade 4	D4T/3TC/NVP	M 60	Ischémique
7	Féminin	35ans	148	Stade 3	D4T/3TC/NVP	M 48	Ischémique

AZT= Zidovudine, 3TC= Lamivudine, D4T= Stavudine, EFV= Efavirenz, NVP= Névirapine

* = CD4 par mm3 à l'initiation du traitement antirétroviral

** = TAR = Traitement aintirétarovial

*** = délai d'apparition de l'AVC après initiation au traitement antirétroviral.

**Tableau 3:** Facteurs associés à la survenue d'un AVC

	Accident vasculaire cérébral	
	Valeur du Khi-2	P-value
Combinaison de TAR	4,625	0,549
Stade SIDA (III et IV) de l'OMS	11,537	**0,001**
CD4 > 100/mm^3^		**0,033**
Circonstances de découverte du VIH		0,101
Charge virale >10.000cpoies/ml		1,000

## Discussion

Dans notre étude, le sexe féminin représente 62,4% des patients contre 37,6% pour les hommes pour un sexe ratio F/H de 1,6. Ceci est le reflet non seulement de la démographie mais aussi de l’épidémiologie de l'infection par le VIH au Cameroun [1]. Nos résultats sont similaires aux 60% de femmes et sexe ratio F/H de 1,5 rapportés en Afrique du Sud par Mochan et al. [10]. L’âge moyen de nos patients étaient de 40,1 ± 9,9 ans, l’étude sud-africaine rapportant 32,1 ans [10]. Une méta-analyse rapporte l’âge moyen des patients à 42,9 ans aux USA et 39,8 ans au Malawi similaire à nos résultats [11]. L'incidence des AVC étaient de 1,7% soit 3 pour 1000 personnes-année dans notre série. L'incidence annuelle des AVC varie entre 1% et 5% [11, 12]. En ce qui concerne les AVC ischémiques, leur incidence est estimée à 5,27 pour 1000 personnes-année [13]. L’âge moyen de survenue de l'AVC était de 48 ans pourtant dans la population globale des patients victimes d'AVC dans le même hôpital, l’âge moyen de survenue est de 58.66 ans [14], soit 10 ans de plus. Ceci peut suggérer que les patients infectés par le VIH sous TAR auraient plus de probabilité de faire un AVC. Ceci serait évidemment le fait des modifications rencontrées chez ces patients: troubles de la crase sanguine avec déficit en protéines S, C, présence d'anticorps anticardiolipines et de complexes immuns [10], la rigidité carotidienne avec perte de la compliance vasculaire à l'origine d'AVC [14]. Les AVC ischémiques représentaient 85,7% des cas dans notre étude. Cette trouvaille concorded'autres résultats rapportés ailleurs. Les deux séries sud-africaines rapportent respectivement 94% et 96% d'AVC ischémique [10, 14] et 96. Les facteurs associés à la survenue d'AVC étaient le stade SIDA de l'OMS et le taux de CD4 > 100/mm^3^. Mochan et al. [10] rapportent comme facteurs étiologiques probables les infections opportunistes du système nerveux central (principalement les méningites), les coagulopathies, les cardiopathies emboligènes et l'hypertension artérielle. Cette étude purement observationnelle descriptive présente des limites puisque porte sur un échantillon relativement faible avec une durée de suivi courte. Des travaux prospectifs comparatifs avec une population témoins faite de personnes non infectés ou infectées sans TAR permettront de bien appréhender les facteurs étiologiques des AVC dans cette population spécifique.

## Conclusion

L'incidence des AVC est faible et survient à un âge précoce et justifie une surveillance accrue des cliniciens sur la prise en charge des facteurs de risque cérébro-vasculaire. Une étude prospective avec une population témoins est nécessaire.

### Etat des connaissances actuelle sur le sujet

- Le VIH accélère l'athérosclérose et peut favoriser les vascularites via les infections opportunistes du système nerveux central;
- Les antirétroviraux sont pourvoyeurs de facteurs de risque de maladie cérébro-vasculaire mais leur incidence reste méconnue dans notre contexte.

### Contribution de notre étude à la connaissance

- Notre étude apporte l'incidence/le taux d'incidence chez les patients VIH positifs traités;
- Elle détermine les facteurs associés à la survenue des AVC chez patients VIH positifs traités par antirétroviraux;
- Elle situe le délai moyen de survenue et le type d'AVC permettant au clinicien de renforcer la surveillance de ces patients.
